# The Paradox of Suicide Prevention

**DOI:** 10.3390/ijerph192214983

**Published:** 2022-11-15

**Authors:** Kathryn Turner, Anthony R. Pisani, Jerneja Sveticic, Nick O’Connor, Sabine Woerwag-Mehta, Kylie Burke, Nicolas J. C. Stapelberg

**Affiliations:** 1 Metro North Mental Health, Metro North Health, Brisbane, QLD 4029, Australia; kylie.burke3@health.qld.gov.au; 2 Center for the Study and Prevention of Suicide, University of Rochester Medical Center, Rochester, NY 14642, USA; anthony_pisani@urmc.rochester.edu; 3 Gold Coast Primary Health Network, Robina, QLD 4226, Australia; jernejas@gcphn.com.au; 4 Clinical Excellence Commission, Sydney, NSW 2065, Australia; nick.oconnor@health.nsw.gov.au; 5 Mental Health and Specialist Services, Gold Coast Hospital and Health Service, Gold Coast, QLD 4215, Australia; sabine.woerwagmehta@health.qld.gov.au (S.W.-M.); chris.stapelberg@health.qld.gov.au (N.J.C.S.); 6 Faculty of Health Sciences and Medicine, Bond University, Gold Coast, QLD 4215, Australia; 7 School of Psychology, The University of Queensland, Brisbane, QLD 4072, Australia; 8 Australian Research Council’s Centre of Excellence for Children and Families over the Life Course, Brisbane, QLD 4068, Australia

**Keywords:** suicide prevention, mental health service, pathway of care

## Abstract

The recognition that we cannot use risk stratification (high, medium, low) to predict suicide or to allocate resources has led to a paradigm shift in suicide prevention efforts. There are challenges in adapting to these new paradigms, including reluctance of clinicians and services to move away from traditional risk categorisations; and conversely, the risk of a pendulum swing in which the focus of care swings from one approach to determining service priority and focus (e.g., diagnosis, formulation, risk and clinical care) to a new focus (e.g., suicide specific and non-clinical care), potentially supplanting the previous approach. This paper argues that the Prevention Paradox provides a useful mental model to support a shift in paradigm, whilst maintaining a balanced approach that incorporates new paradigms within the effective aspects of existing ones. The Prevention Paradox highlights the seemingly paradoxical situation where the greatest burden of disease or death is caused by those at low to moderate risk due their larger numbers. Current planning frameworks and resources do not support successful or sustainable adoption of these new approaches, leading to missed opportunities to prevent suicidal behaviours in healthcare. Adopting systems approaches to suicide prevention, such as the Zero Suicide Framework, implemented in a large mental health service in Australia and presented in this paper as a case study, can support a balanced approach of population- and individual-based suicide prevention efforts. Results demonstrate significant reductions in re-presentations with suicide attempts for consumers receiving this model of care; however, the increasing numbers of placements compromise the capacity of clinical teams to complete all components of standardised pathway of care. This highlights the need for review of resource planning frameworks and ongoing evaluations of the critical aspects of the interventions.

## 1. Introduction

Suicide prevention is a priority for healthcare systems. Public healthcare systems have traditionally focused on identifying mental illness and associated risk factors in those presenting with suicidality and assessing immediate to short-term level of risk. Treatment efforts have focused on treating the mental illness and intervening with those assessed as high risk. This approach has significant limitations. Prediction of who will go on to die by suicide is not possible, and categorical assessments of risk (high, medium, and low) should not be used to predict risk or to determine the resources individuals require for care [1,2,3,4,5]. 

There has been a recent paradigm shift within healthcare systems with a growing number of health services now adopting a systems approach to suicide prevention. Systems approaches, such as the Zero Suicide framework (ZSF) [6,7,8], recognise the multiple elements that impact outcomes and the relationships between those elements, and then design processes or policies that positively influence outcomes [6]. For example, the ZSF recognises that many people who die by suicide do not have a severe or enduring mental illness, and most will not have been identified as high risk in recent contacts. ZSF highlights the need to not only treat underlying causes of presentations, but to also use interventions that specifically target suicidality (e.g., safety planning). It also emphasises the importance of non-clinical aftercare as critical components of a pathway-of-care [6]. Systems approaches, implemented with high fidelity, have shown great promise, as evidenced by Stapelberg and colleagues [7] who demonstrated a 35% reduction in re-presentations for suicide attempts for individuals placed on a Suicide Prevention Pathway across a large mental health service in Australia [7,8].

Sometimes paradigm shifts result in overcorrection. In suicide prevention, there is a risk of a pendulum swing, in which the focus of care pivots from one approach to determining service priority and focus (e.g., diagnosis, risk and clinical care) to a new focus (e.g., suicide-specific and non-clinical care), potentially supplanting the previous approach. We argue that an integrated approach is needed that incorporates new paradigms within effective aspects of existing ones. Implementation must combine strategies to address the new paradigm’s impact on the clinical healthcare system with effective change management. Responding to the risk and impact of suicide across the healthcare system requires a careful balance, incorporating diagnosis, risk and clinical care with suicide-specific and non-clinical care interventions. A recent evaluation of The Way Back Support Service (providing non-clinical aftercare after hospital treated suicide attempts) illustrates this by showing that non-clinical aftercare by itself had limited impact on those presenting with deliberate self-poisoning, indicating the need for evidence-based clinical interventions alongside the non-clinical care [9]. 

Tensions and paradox are common in complex systems. Our natural human inclination is to attempt to resolve tensions, usually in a linear or reductionistic [10]. It is important, when these tensions and paradoxes exist, that we remain open to multiple approaches, responding flexibly to new opportunities [11].

The concept of a ‘Prevention Paradox’ has great utility in understanding and responding to tensions in Suicide Prevention. In this paper, we explore its application within efforts for suicide across clinician-level, service-level and system strategy and planning levels. We present a case study, based on work undertaken by a number of the authors, that draws on the implementation of Zero Suicide Framework/Suicide Prevention Pathway in a clinical setting to demonstrate the prevention paradox at the clinician and service levels and describe implications for national mental health service planning frameworks.

## 2. The Prevention Paradox

Epidemiologist Geoffrey Rose coined the term ‘Prevention Paradox’, [12,13] to describe the situation where in some conditions, the majority of the burden of a disease (the impact of a health problem as measured by indicators such as financial cost, mortality and morbidity) comes from those in the population at low or moderate risk of that disease with only a minority from the high-risk population (the number of whom may be relatively small). This is paradoxical, as it is common to assume that those at highest risk will numerically represent the majority of the burden of the disease. Consider the example of alcohol use. The majority of disease burden in the population arises from moderate use, rather than from heavy use and higher individual risk [14,15] due to the larger proportion of individuals in the population with moderate levels of consumption. The prevention paradox therefore helps to explain why more cases of disease or death could be prevented by targeting the broader population or indicated sub-populations, rather than specifically targeting only those with a high-risk profile.

In situations where the Prevention Paradox holds, there must be a balance of population-oriented approaches that target causes of the incidence in the population with individually-oriented approaches that seek to protect susceptible individuals [16]. Structural prevention efforts, such as organizational procedures and pathways targeting populations or sub-populations and/or interventions targeting the socio-economic environment, may hold greater promise for preventing cases of disease and death [15,17,18].

## 3. Evidence for the Existence of a Paradox in Suicide Prevention

Suicide cannot be reliably predicted, even utilising predictive models of suicide with machine learning approaches [19]. Despite there being known risk factors that place some consumers at extremely high risk of dying by suicide, evidence shows that when formal risk assessments have occurred prior to a suicide, the vast majority were assessed as low risk [20]. One particularly high-risk combination is that of a high lethality attempt alongside a diagnosis of serious mental illness. Having a psychiatric diagnosis has previously been found to increase risk of subsequent suicide attempt or death, with particular concern for those with schizophrenia, bipolar disorder, major depression and eating disorders [21,22,23]. For example, a large-scale Swedish National Register linkage study [24] of 48,649 individuals admitted to hospital after attempted suicide, between 1973 and 1982, who were followed for 21–31 years compared death by suicide across different methods (used as an indication of lethality of attempts) and psychiatric diagnoses. Results showed that higher lethality attempts (by hanging, strangulation, or suffocation) had the worst prognosis, with suicide occurring in 54% of men and 57% of women with, 69% occurring in the first year. Further, having a psychiatric diagnosis resulted in an extremely high risk for those with high lethality attempts. For those with a non-organic psychosis plus a suicide attempt by hanging, 84.1% of men and 84.4% of women died by suicide during follow-up, the majority in the first year [24]. These outcomes reinforce for clinicians the importance of assessing risk, including lethality of attempts and psychiatric diagnosis, in order to identify those at highest risk.

Further examination of data from Runeson et al. [24] reveals that while suicide attempt by hanging carried a 6.2 times higher hazard ratio for subsequent death than low lethality attempts by poisoning, it is in fact the latter cohort that accounted for the largest number of subsequent deaths. Specifically, in the study sample, 83.8% of people presented with an initial suicide attempt by poisoning, and while only 10.5% of those died by suicide during the follow-up period, they accounted for 74.4% of total recorded deaths.

A similar conclusion is evident for suicides by people with psychiatric disorders. Runeson et al. [24] found that particular diagnoses at time of initial attempt or within one week thereof, significantly increased the risk for death by suicide. However, given the majority (73.6%) of people presenting with suicide attempt in this study did not have a psychiatric diagnosis at the time of presentation, their contribution to the total number of observed deaths remained substantial (51.2%). Thus, it is clear that moving from the traditional focus on high risk and mental illness is required if clinicians are to have a greater impact on preventing suicides by consumers seen in health services.

These findings align with the prevention paradox as outlined by Rose [12]. Despite longstanding recognition of the inherent limitations, public mental health services are engineered and resourced around the assumption that ‘high risk’ consumers are those who will make up most of those who subsequently die by suicide, and that the primary responsibility lies in successfully identifying people who have a mental illness and are at high risk, and then intervening with this group [2,25]. This flawed assumption is one that many services and clinicians have been reticent to abandon, resulting in missed opportunities to intervene and prevent suicides that occur in those with lower risk (e.g., low lethality, no psychiatric diagnosis). A re-engineering of our acute mental health services’ responses, including planning and funding for services, is therefore required.

## 4. The Paradox for Individual Clinicians

Suicide prevention is a complex field. Causative factors for suicide include a large array of biological, psychological and social factors, frequently in combination, and with confounding between risks [26,27]. Clinicians must traverse these complex factors and the interplay between them to effectively respond to the challenge of suicide prevention. Suicidality (ideation, intent, suicide attempts and self-harm) is not a condition in itself, but an indication of severe distress within the context of many complex factors. Many who go on to die by suicide have not previously expressed suicidal ideation or intent [28], further confirming the importance of identifying, assessing and intervening for those who do not have suicidal behaviours at presentation, but who have other indicators of risk (e.g., distress, severe depression) or other significant mental illness.

Risk stratification (high/medium/low) cannot predict suicide outcome [29,30,31,32], and contributes little to patient safety. Categorical risk prediction leads to high rates of false positives, resulting in individuals potentially receiving unnecessary treatment, as well as false negatives, resulting in potential denial of necessary treatment [32]. The limited sensitivity of categorical approaches remains pertinent even in studies utilising machine learning algorithms, with up to 25% to 50% of suicides still missed [19]. Categorical risk approaches reinforce a static, cross-sectional assessment of risk, which fails to adequately address the dynamic and fluctuating nature of risk. Nor do they emphasise the importance of addressing modifiable existing factors, while anticipating and mitigating foreseeable future factors that have potential to rapidly increase risk [33,34]. 

Despite these limitations, risk categorisation approaches (in recent years optimised through machine learning approaches), may still flag consumers who would benefit from more targeted bio-psycho-social assessment and facilitate development of an appropriate, individualised care plan [35,36]. However, it remains the case that risk screening tools should not be used to predict risk or decide on the allocation of specific resources, such as level of care [37]. 

### 4.1. Clinician Reluctance for Adopting the Shift in Paradigm

Most clinicians have been trained in categorical approaches to risk assessment and have throughout their career been expected to document risk in these terms. Change to such entrenched practices is challenging. Carter and Spittal [31] noted several contributors to clinicians’ reluctance to change: the publication and endorsement by suicide prevention organizations of a range of tools designed to identify and categorise ‘high risk’ consumers; confirmation bias in clinicians who predominantly categorise consumers as low risk prior to discharge, which due to the low prevalence of suicide will prove to be accurate in the vast majority of cases; and, because ‘low risk’ ratings provide some reassurance and relief from the pressures of scarce resources and escalating demands.

Understanding our healthcare system as a Complex Adaptive System (CAS) [11] provides insight into this reluctance to move away from risk prediction. A CAS comprises “a collection of individual agents with freedom to act in ways that are not always totally predictable, and whose actions are interconnected so that one agent’s actions changes the context for other agents” [11]. An agent’s actions are based on internalised rules, “instincts, constructs, and mental models” [11], although they are not fixed. Braithwaite [38] noted that clinicians either adopt or ignore proposed change initiatives and ideas according to their own logic or local culture. Plsek and Greenhalgh [11] describe the importance of tension and paradox as part of complex systems and warn against reductionist approaches to prematurely resolving these paradoxes. They note the “insoluble paradox between the need for consistent and evidence-based standards of care and the unique predicament, context, priorities, and choices of the individual patient” [11].

We argue that embracing the Paradox of Prevention provides a useful mental model for clinicians to assist with balancing an individualised approach to care with use of standardised structural prevention efforts, (e.g., development of organizational care pathways for groups of patients) regardless of individual risk or diagnosis. Placement on a pathway, combining individualised engagement, assessment of modifiable risk factors and a tailored, collaboratively developed care plan within a model of care that provides interventions for *all* hospital presenting patients, offers a balanced approach that can consolidate the individual and population-based suicide prevention focus. Carter et al. [32] describe groups of patients benefiting from these pathways of care as ‘unselected’ hospital treated self-harm patients.

### 4.2. Reasons for Low-Risk Rating Prior to Suicide

As already noted, most people who were assessed prior to suicide have been categorised as ‘low risk’ [20]. Although the prevention paradox may help to explain this finding, there may be other contributors. For example, individuals may not disclose intent due to factors such as shame, stigma or not wanting to be a burden [39]. Further, suicidality is fluctuating by nature, which means that while suicidal intent may have resolved at the time of assessment, it may rapidly re-escalate under certain conditions [40]. Additionally, most consumers presenting to emergency departments are likely to be at a higher risk than the general population resulting in clinicians being repeatedly exposed to suicidality and the indicators associated with higher lethality attempts. Over time, this can result in reduced situational and contextual awareness and desensitisation to and underestimation of risk. Finally, healthcare systems are resource scarce environments and thus there may be significant pressure to label someone ‘low risk’ if the decision is to discharge them.

### 4.3. Addressing the Paradox at a Clinician Level

To effectively address the Paradox of Suicide Prevention, it is vital that at the clinician level, a focus is retained on best evidence in individualised psychosocial assessment, diagnosis, formulation, addressing needs, clinical treatment, and clinical handover. Additionally, clinicians must embrace best practice in suicide specific care, along with the benefits of a population-based structure (e.g., a pathway of care that incudes safety planning, counselling on access to lethal means, and rapid follow-up aftercare in the community). This balanced approach must be implemented with all consumers presenting with suicide attempts or suicide crisis, irrespective of the diagnosis or risk category.

It is important to note that adoption of a standardised pathway approach is likely to impact large numbers of people who may not benefit from an intervention (given that many in this broader cohort of patients will never develop further suicidal behaviour). This underlines the importance of ensuring that clinicians understand the role of population-based approaches in prevention of suicide. Without this understanding, there is likely to be negative impacts on clinician motivation to adopt the approach [13,41]. We propose that the prevention paradox provides a useful ‘mental model’ to support clinicians (and services) with this understanding.

## 5. The Paradox for Public Mental Health Services

At a service level, addressing the prevention paradox has enormous implications in terms of broadening the numbers of consumers who become ‘in scope’ for intervention and follow-up. Services can accomplish this through supporting both an individualised formulative risk mitigation/prevention approach and the development of structural prevention efforts, such as procedures and pathways that can support interventions for populations or sub-populations of consumers presenting to services, regardless of an individual assessment of risk. Despite the merits of this approach as a prevention and treatment strategy, it must be recognised that this comprehensive and balanced approach places pressure on resources that were previously reserved for the smaller cohort of ‘high risk’ consumers. As will be described in a case study below, when this paradox is addressed within services, good fidelity to new models of care can achieve good outcomes for individuals, yet also rapidly place substantial strain on government funded public mental health services to achieve the needed fidelity, safety and quality of care, and risk overwhelming them.

Year on year, significant increases in suicidal presentations in services that outstripped population growth (for a recent study in Queensland, see [42]), in the absence of proportionate increase in funding to clinical services, has led to increasing strain on services [43,44]. Demands placed on services through a combination of increased presentations and addressing the prevention paradox have not been resourced. Despite these resource limitations, services must maintain a focus on the importance of providing interventions for the broader cohort of patients, while reinforcing, and providing training and ongoing supervision for best practice clinical care that delivers comprehensive care, formulation, diagnosis and treatment of mental illness and substance use disorders.

## 6. The Paradox at a System Planning and Strategy Level

Suicide is a complex and challenging issue with no single solution to prevent or reduce its impact. Suicide prevention requires a whole-of-government, cross-sectoral approach to planning, strategic policy development and service delivery [45,46]. At a system strategy and planning level, there is a risk of underestimating complexity and there must be recognition of the tensions and paradoxes described here. Funding is required to support care at all levels: upstream interventions addressing the social determinants of suicide; clinical and non-clinical early intervention solutions for preventing crises; non-clinical supports for aftercare; and identification and treatment of people who have attempted or are contemplating suicide with/or without serious mental illness.

Health service funding and the resulting capacity to respond to suicidal presentations are linked to planning frameworks. The development of planning frameworks is a lengthy process, often requiring several years. Unless the models of care informing our Frameworks are continually updated, new models of care such the Zero Suicide in Healthcare in Queensland [47], or Towards Zero in New South Wales [48], and other new models that might arise from, for example, the Victorian Royal Commission into Mental Health Services [49], will not be adequately resourced.

The Australian National Mental Health Services Planning Framework (NMHSPF) is one example of an integrated planning tool that estimates the resources required to deliver the optimal mix of mental health services to a population [50]. In Australia, it is critical that the NMHSPF, which currently does not plan for suicide and crisis, let alone the implications of recent paradigm shifts, is revised to take account of, and recognise, suicide and crisis, as well as the shift in paradigm impacting service activity, and increases in presentations. Without this, there is a risk that funding will inadequately serve both those presenting with suicidality and crisis and those with severe and complex mental illness.

## 7. Addressing the Prevention Paradox

Addressing the prevention paradox requires systems planning and resourcing to support services, teams, and clinicians to embrace both traditional individualised risk identification and management approaches, and broader population approaches. This ambidextrous approach, which addresses both aspects of the paradox and tensions, is an important capability of resilient organizations, particularly in high-risk areas which require both stability and flexibility [51].

### 7.1. Unintended Consequences and Challenges

As with all actions within complex systems, there are risks of unintended consequences and unpredictable outcomes. One risk associated with the prevention paradox is that a shift to targeting the larger group of people with lower risk may reduce responsiveness to those at higher risk and jeopardise their care [52]. This highlights the challenges of embracing complexity and paradox, and the need to combine individualised approaches with population-based approaches to care.

### 7.2. Balancing Individual Risk Identification and Management with Population-Based Suicide Prevention Risk Reduction in Mental Health Services

We propose that population-based efforts within the mental health service system combine ‘selective’ interventions targeted to broad groups who as a population have increased risk, and ‘indicated’ interventions that target individuals with specific risks, for example, through diagnosis or lethality of attempts [26].

**Figure 1:**
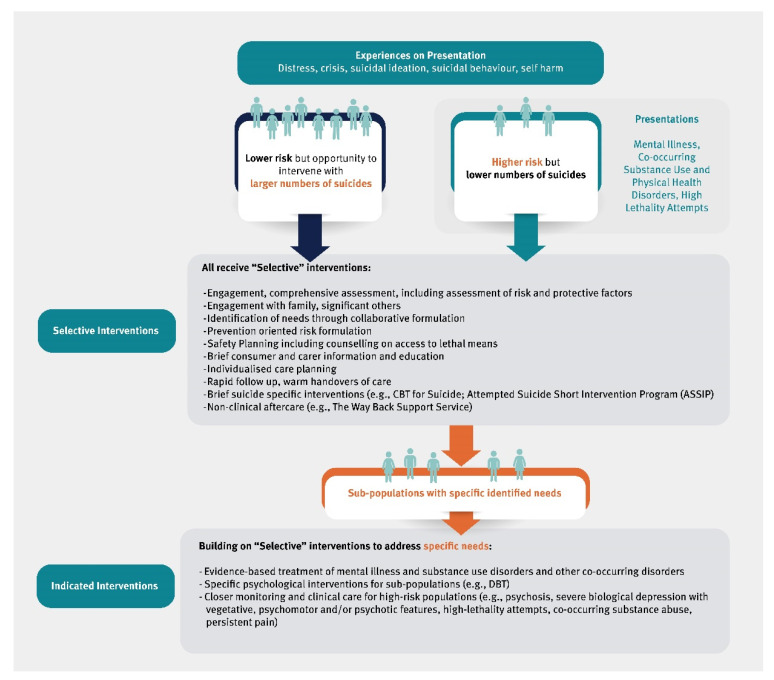
Selected and Indicated Populations and Interventions for Suicide Risk in Healthcare.

Figure 1 describes each group and the types of interventions that would be provided in this model. People presenting to services in distress or as suicidal would be provided with ‘selected’ interventions; and those who are also presenting with mental illness, co-occurring disorder, or features of higher risk such as high lethality attempts would be provided with ‘indicated’ interventions in addition to the ‘selected’ interventions in a combined approach.

The Case Study below illustrates outcomes and learnings from a health service that adopted a combined individualised risk or needs identification and management approach within a population-based standardised pathway of care approach to addressing the paradox in suicide prevention. We discuss the resulting tensions, strategies for resolving these tensions, and the implications for national planning frameworks.

## 8. A Case Study: The Gold Coast Suicide Prevention Pathway

Gold Coast Mental Health and Specialist Services (GCMHSS) in Queensland, Australia, implemented a Suicide Prevention Strategy (SPS) based on the Zero Suicide Framework (ZSF) in 2016 [8]. The ZSF is a systems approach to suicide prevention in a health service with a focus on leadership, training and support for staff, inclusion of lived experience, cultural change, based on principles of restorative just culture [53], a pathway of care and a continuous quality improvement approach. GCMHSS’s implementation of ZSF included the development of a clinical Suicide Prevention Pathway (SPP) and upskilling of clinical staff that moved the service away from risk prediction approaches and use of diagnosis as a gateway to care. SPP includes a number of suicide specific interventions, such as safety planning, counselling on access to lethal means, consumer and carer psycho-education, and face-to-face contact with a mental health clinician within 48 h of discharge [8]. One aim was rapid connection of those who do not require ongoing public mental health support to other community support services, including primary care, private sector and non-clinical aftercare services, such as The Way Back Support Service (a not-for-profit organization providing non-clinical psychosocial support and transition services for individuals who have attempted or are at risk of suicide) [9]. High quality clinical handover in this transition phase was emphasised.

The SPP supports engagement and development of a therapeutic relationship, promoting comprehensive assessment and an exploration of suicidal intent using the Chronological Assessment of Suicide Events (CASE), and Prevention Oriented Risk Formulation [54]. These resources are designed to facilitate individualised care plans that address the challenges of situational and contextual awareness associated with repeated exposure to suicidal presentations. The SPP sets minimum expectations for a standardised assessment and treatment pathway, while allowing flexible tailoring to ensure that the care plan meets an individual’s clinical needs.

To address the need for more individualised assessment and treatment, while balancing the tensions of service demand and capacity, the GCMHSS developed a set of criteria for placement on the SPP that covered a sub-population of ‘unselected’ consumers and reduced the reliance on individual clinician assessment of risk when determining intervention needs. Criteria for being placed on the pathway included: all of those who presented with a recent suicide attempt, or with suicidal thoughts and a past history of suicide attempt, and all consumers admitted to an inpatient unit with suicide risk (regardless of presence or absence of a mental health diagnosis, lethality of the attempt or perceived level of individual risk). Clinicians still retained discretion to add others who did not meet those criteria. These criteria broadened the scope for who should receive assessment and intervention, resulting in many more consumers being eligible for at least brief interventions and follow-up.

**Figure 2:**
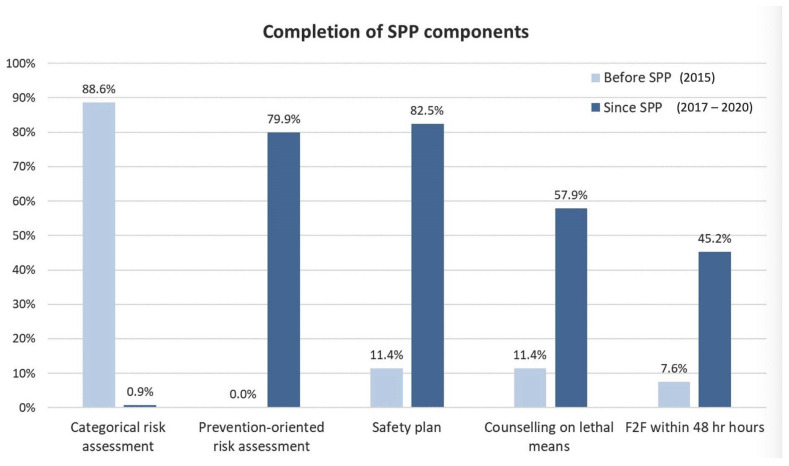
Completion of SPP components.

The SPP was rapidly adopted, with good fidelity to the pathway’s components. Figure 2 demonstrates the extent to which GCMHSS was successful in adoption of a prevention-oriented risk assessment, safety planning and counselling on access to lethal means. Notably, prevention-oriented risk assessment had replaced categorical risk assessment within the first few months post-implementation and a marked increase in face to face follow-up within 48 h of discharge had also occurred [8].

An important component of the SPP was the incorporation of a strategy for continuous monitoring of the processes and impacts that enable opportunities for reflecting, learning and iteratively improving the system. This identified several challenges and resulted in the design and implementation of strategies to counter them. A number of these are outlined below.

### 8.1. Challenges: Indicated versus Selective Approaches

A year into the implementation of the SPP, a number of issues were identified. In terms of a universal move away from categorical risk prediction approaches, it was apparent that, while this transition was well supported by the use of the Prevention Oriented Risk Formulation for consumers who were being placed on the SPP, it was not being adopted universally for all consumers, in part due to its primary focus on suicide risk. It was felt that adapting the Prevention Oriented Risk Formulation to address multiple domains of risk (suicide, violence, vulnerability) would support its utility as a universal strategy.

There were also some concerns that there was more emphasis on suicide specific and population-based approaches to the pathway, with a potential de-emphasis of important clinical aspects of diagnosis, formulation and clinical treatment. Work was therefore undertaken to strengthen individualised consideration of formulation and diagnosis.

These two issues resulted in the development of the “Integrated Formulation” (IF) (IF) [55]. The IF builds on the atheoretical “5 Ps” approach to formulation (Presenting, Predisposing, Precipitating, Perpetuating and Protective factors) and provides support for a collaboratively developed [56], shared understanding of the unique circumstances for the consumer. The IF builds understanding around why consumers have presented in this way at this time and understanding of the strengths and goals of the consumer. It supports a reflection on diagnoses to challenge cognitive bias, including encouragement of clinicians to pause and reflect on questions regarding diagnosis and co-occurring disorders, the meaning of risk factors to the individual, and countertransference issues. The IF assists the clinician to consider risk factors across overlapping and interacting domains of violence, suicide and vulnerability, and is influenced by theoretical frameworks for suicide such as the Integrated Motivation Volitional Model for suicidal behaviour [57]. Central to the IF is the use of the Prevention Oriented Risk Formulation [54], adapted for multiple domains of risk (suicide, violence, vulnerability), articulating four components: risk status relative to others, risk state relative to self, availability of resources and foreseeable changes. The result is an individualised care plan, which includes plans to address modifiable risk factors, provision of evidence-based treatment for mental illness and co-occurring disorders, and contingency plans to address foreseeable changes that may impact future suicidal ideation and behaviour. Adapting the Prevention Oriented Risk Formulation from a focus on suicide to incorporate multiple domains of risk supports its more universal application across the service.

### 8.2. Challenges: Resource Implications

**Figure 3:**
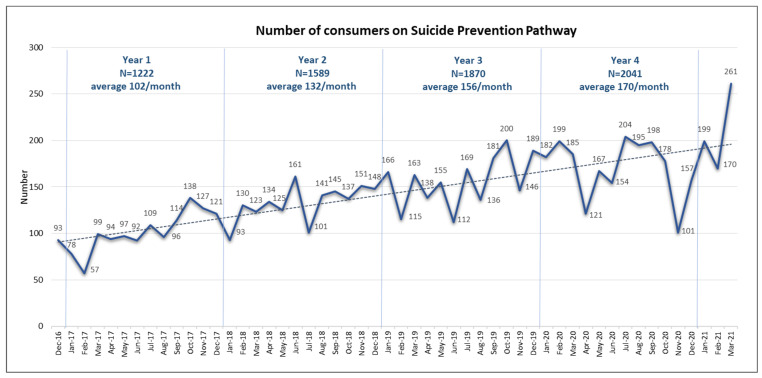
Numbers of consumers on Suicide Prevention Pathway at Gold Coast Mental Health and Specialist Services.

Due to the increasing number of suicidal presentations to Gold Coast Emergency Departments (with average annual increase of 13% [42] and the increased uptake of the SPP, the number of consumers being placed on the SPP gradually increased over time. In total, there were 7445 consumers placed on the pathway between December 2016 and March 2021, with average numbers on the pathway almost doubling over 4 years (Figure 3). This outcome demonstrates the utility and success of the implementation of the approach; however, it also highlights the considerable resource requirements for maintaining quality and fidelity over time.

The rates of completion of individual components of SPP, shown in Figure 2, are encouraging and a result of concentrated efforts placed on training and supporting staff across all service lines, before, during and after the implementation of the ZS framework. However, during this period, considerable fluctuations in fidelity have also been observed, which seemed aligned with the variations in the numbers of consumers placed on SPP.

Table 1 below shows the correlations between the completion of individual SPP components and the number of SPP consumers managed by the Acute Care Team (ACT) between January 2018–December 2020. ACT provides care to around 75% of all SPP placements, accounting for an average of 120 consumers per month; therefore, their capacity to maintain high fidelity to the SPP model is likely to be most impacted by the increasing demand.

There were significant negative associations between the number of individuals on the pathway and the SPP components of safety plan, follow-up and care-plan components. The correlations were moderate and negative indicating that as the number of SPP placements increased the completion of SPP components decreased. We did not see a reduction in fidelity to other aspects of our model, including the Prevention Oriented Risk Formulation, the CASE approach or counseling on lethal means.

The SPP represents a minimal suite of interventions that aligns with the principles of population-based interventions, are respectful of the rights of the individual [41], and are low cost, effective, short term, with few potential negative impacts [32]. Despite this, SPP has demonstrated positive impacts for consumers showing reductions in risk of re-presentation with a suicide attempt by 35%, regardless of frequency of presentations, indigenous status, age or diagnosis of a personality disorder [42]. The rate of suicides of GCMHSS consumers, reported as a 3-year rolling average, has also demonstrated a trending down and reduction of 23% since the implementation of SPP, although given the relatively low numbers of deaths by suicides this finding needs to be interpreted cautiously [8].

The SPP is a relatively light-touch, minimal and effective approach that can be successfully deployed within a public mental health service. However, the significant associations between increased numbers of suicidal presentations and people placed on the pathway and reduced fidelity demonstrate that maintaining successful implementation within the existing clinical resources is a substantial challenge. If we are to make a sustained difference to the prevention of suicide, the policy and funding frameworks that guide the planning of mental health services to incorporate these new paradigms, as well as commensurate resourcing, must be prioritised.

### 8.3. Implications for Planning Frameworks

While the extent to which investments in mental health service capacity will contribute to reductions in suicide is unknown, there is some evidence that staffing, training, capacity of community mental health services, specialist assessment capacity and keeping consumers engaged in services all matter [58,59]. Moreover, there may be some harm (i.e., increased suicides) if an increased number of people are identified as needing specialist suicide prevention interventions and these interventions are not within the capacity of clinical services [60].

## 9. Conclusions

Complexity surrounding suicide risk assessment and management has led to the need for a paradigm shift in mental health services that addresses the prevention paradox. Pursuit of balance between individualised risk and needs identification and management approaches, and population-based structural prevention efforts supported by standardised pathways of care, is critical for clinicians who provide care and for services charged with developing effective supportive care pathways. The prevention paradox provides leaders within services and individual clinicians with a useful mental model to guide this work, supporting an ambidextrous approach by simultaneously adopting more population-based efforts and individualised responses when engaging with the person in distress. Systems frameworks such as the Zero Suicide framework and pathways of care can address many of the identified issues and lead to meaningful improvements in clinical care services [7,61].

This paradigm shift does, however, bring with it challenges. As with all complex health systems which aspire to resilient health care, there is a need for the ability to anticipate developments, respond to problems, monitor processes, and learn from experience [62]. This also aligns well with the Zero Suicide framework, which embeds an expectation of continuous quality improvement, and a relentless pursuit of zero suicides through iterative improvements. The GCMHSS case study demonstrates that it is possible to achieve this paradigm shift, yet there are challenges that need to be addressed if such a shift is to be implemented with fidelity and sustained over time. The IF provides an approach to ensure a person-centred, strengths-based, individualised approach to understanding the unique aspects of the person at risk of suicide, and to engage in collaborative individualised care, within a wider context of a standardised pathway of care.

There remain ongoing tensions between population-based and individualised risk identification approaches to care, at both a service level and individual patient level, and a need to constantly monitor and respond when there is too much of one over the other, and where unintended consequences may emerge.

The resource implications of this important change in paradigm are significant. Figures provided for GCMHSS indicate that capacity has now been reached within the Acute Care Team, which provides care for the majority of consumers on the SPP, and this impacts on fidelity of the standardised pathway of care. It is noteworthy that the SPP elements for which the completion seems most dependent on the demand experienced by the service are the same ones for which exists most robust evidence-base of efficacy (safety planning intervention [63]; rapid follow-ups [64]; and fidelity to the ZSF [61]). There is a risk that reductions in fidelity to the SPP will lead to reduced impact, increased suicide attempt re-presentations and compromise the opportunity to save lives. While there is work currently being undertaken at GCMHSS to explore which components of the SPP are most associated with positive outcomes, currently this is not known.

This paradigm shift clearly requires extra resourcing to keep up with the demand. A review of planning frameworks such as Australia’s NMHSPF is critically required, with some urgency, to determine appropriate levels of resourcing to address suicide and crisis within mental health services. Furthermore, it is essential that addressing significant needs in suicide and crisis must not come at the expense of consumers with severe and complex mental illness.

## Figures and Tables

**Table 1:** Pearson correlation coefficient between number of SPP placements and completion of SPP components by the Acute Care Team.

SPP Component	Number of Consumers on SPP
Prevention-oriented risk formulation	−0.026
CASE approach	−0.126
Safety plan	−0.476 *
Counselling on lethal means	−0.002
Follow-up in 48 h	−0.459 *
Care plan	−0.502 **

Note: * *p* < 0.05; ** *p* < 0.001.

## Data Availability

Due to their sensitivity, data cannot be made available for the case study part of this paper. However, corresponding author can be contacted for any additional information around data collection and analyses.
